# Curative remnant total pancreatectomy for recurrent pancreatic acinar cell carcinoma: A case report

**DOI:** 10.1016/j.ijscr.2022.107091

**Published:** 2022-04-18

**Authors:** Masahide Hiyoshi, Kengo Kai, Takeomi Hamada, Koichi Yano, Naoya Imamura, Atsushi Nanashima

**Affiliations:** Division of Hepato-Biliary-Pancreatic Surgery, Department of Surgery, University of Miyazaki, Faculty of Medicine, Miyazaki, Japan

**Keywords:** Pancreatic acinar cell carcinoma, Recurrence, Total pancreatectomy, Neoadjuvant chemotherapy, Case report

## Abstract

**Introduction and importance:**

Acinar cell carcinoma (ACC) of the pancreas is a rare pancreatic cancer subtype (incidence, 0.5–2%) with unclear epidemiology and prognosis. Sometimes, repeat resection including total pancreatectomy is required for recurrence. We report a case of ACC recurring in the remnant pancreatic head after distal pancreatectomy that was successfully cured by remnant pancreatic resection following combination therapy with nab-paclitaxel (nab-PTX) and gemcitabine (GEM).

**Case presentation:**

A 64-year-old woman was referred for pancreatic tumour treatment. CT revealed a 46-mm tumour in the pancreatic body, and endoscopic ultrasonography-guided fine needle aspiration (EUS-FNA) indicated ACC. Distal pancreatectomy was performed, and the final diagnosis was ACC. CT 18 months later showed a 34-mm tumour in the remnant pancreatic head revealed as ACC by EUS-FNA. Portal vein invasion was apparent, so neoadjuvant chemotherapy with nab-PTX and GEM was administered, and remnant pancreatic resection (total pancreatectomy) was performed. No recurrence or distant metastasis was present more than 6 months later.

**Clinical discussion:**

Mean survival time for ACC is 18–47 months, and prognosis is good compared with pancreatic ductal adenocarcinoma (PDAC). ACC tends to extend and grow along the main pancreatic duct, which increases the recurrence rate to 50–60%. EUS and EUS-FNA were useful for evaluating tumour extension and diagnosis. Repeat pancreatic resection that included total pancreatectomy was feasible and could be performed safely.

**Conclusion:**

ACC has a better prognosis than PDAC but with a higher recurrence rate. Aggressive surgical resection that included remnant total pancreatotomy with chemotherapy was useful in treating ACC.

## Highlights

• Acinar cell carcinoma (ACC) of the pancreas is a rare type of pancreatic tumour.
• ACC has good prognosis compared with pancreatic ductal adenocarcinoma, but the recurrence rate is high.
• Surgical resection that includes remnant pancreatic resection is useful for pancreatic tumours.
• Neoadjuvant and adjuvant chemotherapy are useful in treating ACC.

## 1 Introduction

Acinar cell carcinoma (ACC) of the pancreas is a rare subtype of pancreatic cancer whose epidemiology and prognosis have not yet been completely elucidated [1], [2]. Surgery remains the main curative treatment for pancreatic cancer, and sometimes, repeat resection is needed for recurrence. Recently, total pancreatectomy including remnant pancreatic resection has been aggressively performed [3], [4]. Improvement with surgery and powerful combination chemotherapy often dramatically decreases the extension of pancreatic tumours [5].

We experienced a patient with recurrence of ACC that was curatively resected by remnant total pancreatectomy after combination chemotherapy with nab-paclitaxel (nab-PTX) and gemcitabine (GEM). We report and discuss the treatment that included aggressive repeat curative resection and chemotherapy for ACC. This case is reported according to the SCARE 2020 criteria [6].

## 2 Case presentation

A 64-year-old woman was referred to our hospital for further examination and treatment of a pancreatic tumour found by chest computed tomography (CT) performed at a medical check-up. A pancreatic tumour was identified in images obtained near the end of the chest CT imaging protocol. She had no symptoms related to the pancreatic tumour and had no past history or family history except for hypertension.

**Fig. 1:**
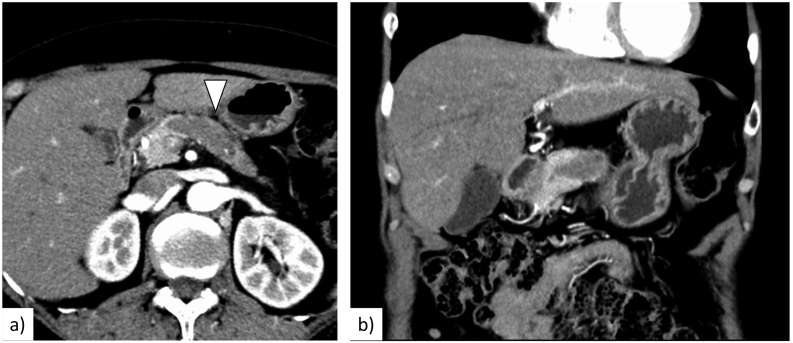
a. An enhanced computed tomography scan revealed a 46-mm low-density mass in the pancreatic body to tail (arrowhead). b. The tumour extended along the main pancreatic duct.

An abdominal CT scan showed a 46-mm low-density mass in the pancreatic body and dilatation of the distal main pancreatic duct (MPD) (Fig. 1). The tumour appeared to be localized and extend along the MPD. Endoscopic ultrasonography (EUS) showed a 20.6 × 12.3-mm hypoechoic mass in the pancreatic body. EUS-guided fine needle aspiration (EUS-FNA) revealed polygonal cancer cells proliferating in a fused tubular and papillary pattern. Immunohistochemistry was positive for trypsin and BCL-10, indicating that the pancreatic tumour was ACC. Tumour markers such as carcinoembryonic antigen, carbohydrate antigen 19-9, DUPAN-2, and SPan-1 were not elevated. Fluorodeoxyglucose-positron emission tomography showed positive uptake in the pancreatic tumour but no distant metastasis.

We performed radical antegrade modular pancreatosplenectomy with lymph node dissection for the pancreatic body tumour. Although the pancreatic cut margin was close to the tumour, intraoperative frozen section revealed no malignant tissue at the transected pancreatic parenchyma.

**Fig. 2:**
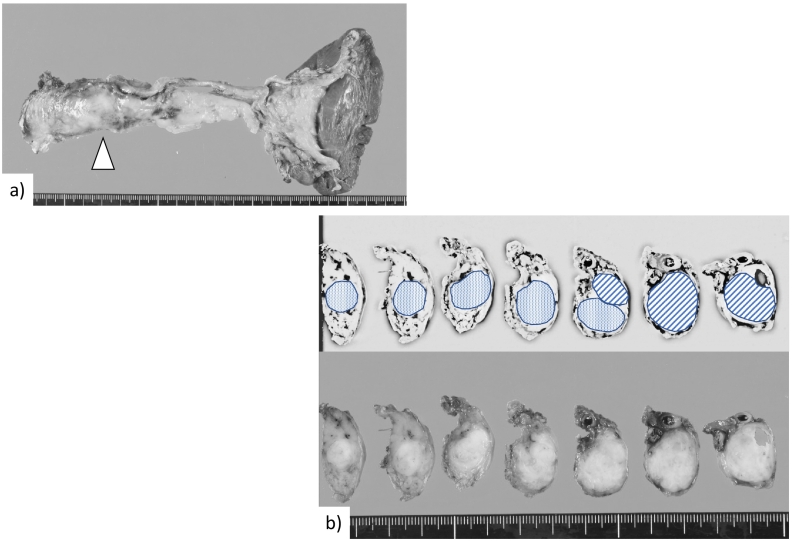
Macroscopic photos of the excised tumour. a. Distal pancreatectomy was performed. The main tumour was located in the pancreatic body (arrowhead). b. The resected specimen showed a tumour in the pancreatic body (diagonal lines) that extended along the main pancreatic duct (small dots).

Macroscopic findings showed a 30-mm tumour in the pancreatic body extending 30 mm along the inside of the MPD (Fig. 2). Microscopic findings revealed cancer cell invasion into the pancreatic stroma, perineural site, and vessels. The surgical margin was negative, and there was no lymph node metastasis or tumour invasion around the vessels, indicating that R0 surgical resection was achieved.

A postoperative pancreatic fistula was observed that caused a pseudoaneurysm in the gastroduodenal artery (GDA). Although post-pancreatectomy haemorrhage due to rupture of this pseudoaneurysm occurred, transcatheter arterial embolization (TAE) stopped the haemorrhage, after which the patient's condition eventually improved and she was discharged. Adjuvant chemotherapy using S1 was administered but was stopped because of diarrhoea at 11 weeks after start the adjuvant chemotherapy.

She received a regular check-up in our hospital every 3 months with abdominal CT. Follow-up CT performed 18 months after surgery revealed a tumour 34 mm in size in the remnant pancreas that appeared to grow along the MPD and invade the portal vein. EUS showed a 20-mm hypoechoic mass, and EUS-FNA revealed cancer cells that were immunoreactive positive for BCL-10 and trypsin. Tumour markers were not elevated. Considering her past history and pathological findings, we diagnosed recurrence of ACC.

**Fig. 3:**
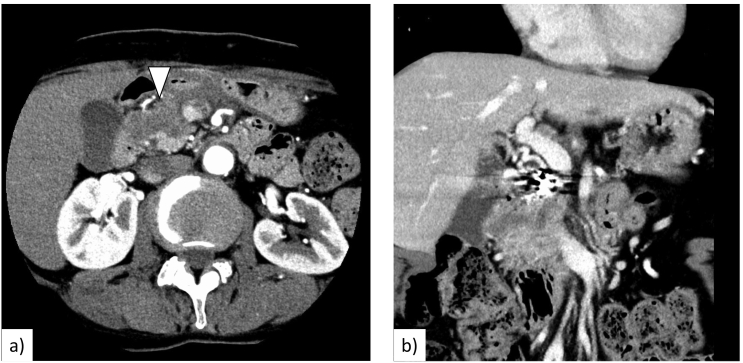
a. A CT scan after chemotherapy revealed that the pancreatic mass had shrunk to 29 mm (arrowhead). b. The border between the tumour and portal vein was unclear.

We administered full-dose neoadjuvant chemotherapy of nab-PTX (125 mg/m^2^) in combination with GEM (1000 mg/m^2^) on days 1, 8, and 15 every 4 weeks for 2 cycles to shrink the tumour and reduce its extension into the portal vein. During chemotherapy, grade 2 bone marrow suppression and grade 1 symptomatic adverse events such as general fatigue, peripheral neuropathy, hair loss, and diarrhoea based on Common Terminology Criteria for Adverse Events (CTCAE) version 4.0 [7] were observed. CT after neoadjuvant chemotherapy revealed that the pancreatic mass had shrunk to 29 mm, and neither distant metastasis nor new lesions were observed (Fig. 3). The effect of neoadjuvant chemotherapy was evaluated as stable disease based on Response Evaluation Criteria in Solid Tumors (RECIST), version 1.1 [8].

**Fig. 4:**
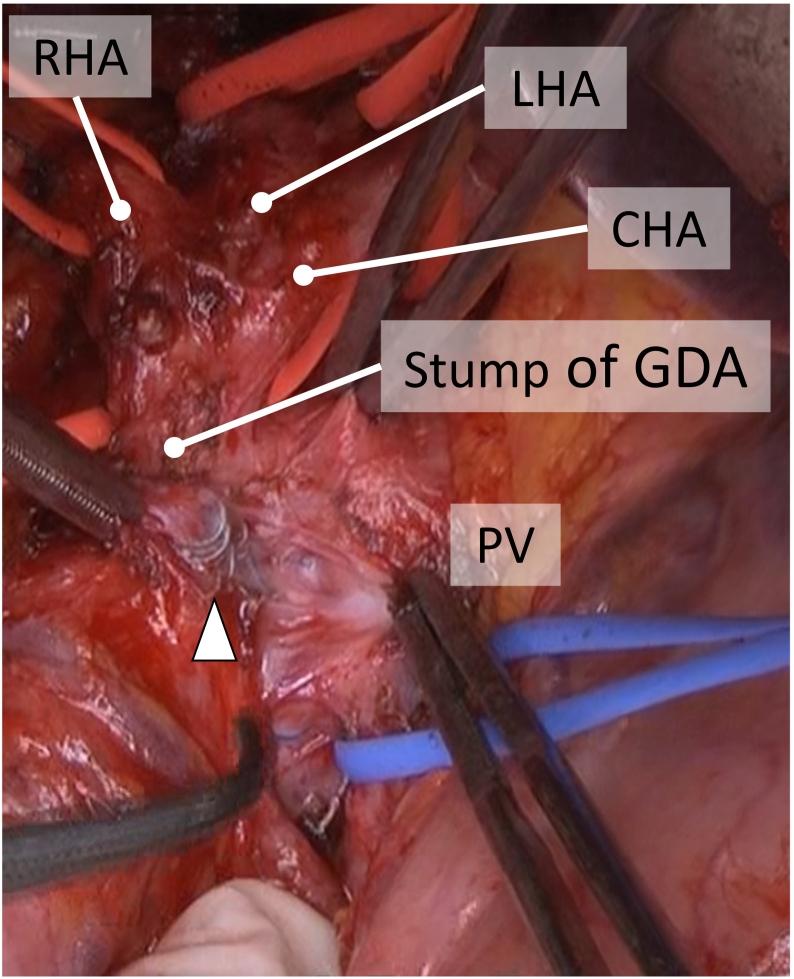
a. Dense adhesions around the stump of the GDA and PV were observed. The coils that were used for arterial embolization were confirmed (arrowhead). GDA: gastroduodenal artery, CHA: common hepatic artery, RHA: right hepatic artery, LHA: left hepatic artery, PV: portal vein.

Four weeks after neoadjuvant chemotherapy, we performed total pancreatectomy as curative resection for the remnant pancreatic head. Although dense adhesions especially around the TAE-treated GDA and the portal vein were recognized (Fig. 4), we removed the coil from the GDA and closed its stump with interrupted sutures using 5-0 monofilament suture. Hepaticojejunostomy was performed by interrupted suture using 5-0 PDS II. Gastrojejunostomy and Braun's anastomosis were performed with a linear stapler. The portal vein was tumour free and did not require combined resection, so we achieved curative resection. On postoperative day 1, she complained of melena without abdominal pain or nausea. Emergent gastrointestinal endoscopy revealed oozing at the gastrojejunostomy site, and haemostasis was performed. Thereafter, no new events occurred and she was discharged.

**Fig. 5:**
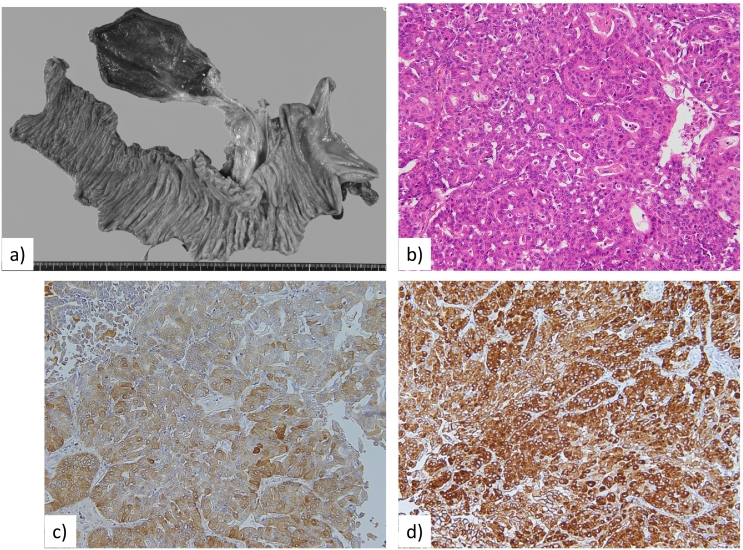
Macroscopic and microscopic photos of the excised tumour. a. Remnant pancreatic head resection (total pancreatectomy) was performed. b. Histological findings with hematoxylin and eosin stain showed atypical cuboidal to columnar cells with hyperchromatic nuclei in an acinar pattern. c, d. Immunohistochemistry showed positivity for trypsin (c) and BCL-10 (d).

Macroscopic findings showed a 50-mm tumour in the remnant pancreatic head. Microscopic findings revealed that cancer cells had invaded the stroma, perineural site, and vessels, and the tumour had changed to grade I fibrous tissue by the Evans classification. Immunohistochemistry was positive for trypsin and BCL-10, indicating the pancreatic tumour to be ACC (Fig. 5). The surgical margin was negative, and no perivascular lymph node metastasis or tumour invasion was present, so we determined that R0 surgical resection was achieved.

The patient has received no adjuvant treatment, and at more than 6 months after surgery, there has been no recurrence or distant metastasis and no elevation of tumour markers.

## 3 Discussion

ACC of the pancreas is a relatively rare malignant tumour with an incidence of 0.5–2% among pancreatic neoplasms [1], [2]. ACC appears to occur as larger tumours in younger patients but causes fewer symptoms such as obstructive jaundice compared with pancreatic ductal adenocarcinoma (PDAC) [9], [10]. Lipase hypersecretion syndrome with cutaneous manifestations often appears and is characteristic in ACC [11]. Commonly, the mean survival time with ACC is 18–47 months, and the prognosis is thought to be better than that for PDAC [9], [12]. However, some authors are noting that the prognosis is becoming worse nowadays [13]. There is no helpful specialized treatment strategy for ACC, and surgical resection is being recommended for resectable cases such as those with PDAC.

Some authors have reported that ACC has a high rate of recurrence of 50–60% [9], [14]. In relation to the recurrence pattern, Wang et al. reported that 75% of patients had local recurrence [9], whereas Seo et al. reported that 100% had distant metastasis [14], and no standard form of recurrence has been confirmed. In addition, ACC tends to extend and grow along the MPD, and EUS can be useful to evaluate tumour extension [15]. EUS-FNA in combination with an immunobiological study with BCL-10 can increase the rate of accuracy of the preoperative diagnosis of ACC [16] as in the present case. Considering that ACC tends to spread along the MPD, we cannot deny that the recurrence formed near the resected edge in the present case even if that edge was free from cancer cells at the initial surgery performed for ACC in the pancreatic body. Ikeda et al. reported multiple occurrences of ACC including synchronous and metachronous types [17]. Although no other lesions were evident by imaging at the initial surgery in the present case, we could not completely deny the possibility of multiple lesions.

Yamada et al. reported that repeat pancreatic resection was useful for pancreatic cancer recurrence, which has an extremely low mortality rate of 1% [3]. Although the present patient had dense adhesions from previous surgery and a ruptured pseudoaneurysm, we achieved complete surgical resection at the repeat surgery, and she was discharged safely. Improvements in surgical technical planning with detailed assessment of anatomy using 3-dimensional CT, perioperative management especially using various insulin formulations, and surgical instruments have allowed the safe performance of aggressive remnant pancreatic resection, i.e., total pancreatectomy. However, clinicians should note deficiencies of exocrine and endocrine function after total pancreatectomy. The development and use of synthetic insulin and supplementation of pancreatic enzymes have resulted in an acceptable quality of life after total pancreatectomy [18].

The present patient was administered S1 as adjuvant therapy. Patel et al. reported that adjuvant systemic adjuvant chemotherapy was beneficial for resected ACC [19]. Recently, neoadjuvant chemotherapy for pancreatic cancer is being actively performed. We used nab-PTX with GEM as neoadjuvant therapy for ACC with suspected portal vein invasion in our patient. Some authors reported that combination treatment with nab-PTX plus GEM showed a favourable tumour-reducing effect and was not associated with severe adverse events for locally advanced unresectable pancreatic cancer [20]. Although there remains no specialized treatment for ACC, as with PDAC, the combination of neoadjuvant chemotherapy, radical surgical resection, and adjuvant chemotherapy was useful in treating ACC.

## 4 Conclusion

The etiology, nature, and prognosis of ACC remain unclear. Although it appears to have a better prognosis than that of PDAC, the recurrence rate is higher. As with PDAC, aggressive surgical resection that included remnant total pancreatotomy was useful in treating the present patient with ACC, and neoadjuvant and adjuvant chemotherapy were also beneficial.

## Provenance and peer review

Not commissioned, externally peer-reviewed.

## Sources of funding

This case report did not receive any specific grant from funding agencies in the public, commercial, or not-for-profit sectors.

## Ethical approval

Our institution determined that this case report was exempt from the requirement for ethical approval.

## Consent

Written informed consent was obtained from the patient for publication of this case report and accompanying images. A copy of the written consent is available for review by the Editor-in-Chief of this journal on request.

## Author contributions

Masahide Hiyoshi performed the second operation and was the main author of the paper. Kengo Kai, Takeomi Hamada, Koichi Yano, Naoya Imamura, and Atsushi Nanashima contributed support during the operations and perioperative management.

## Research registration

Not applicable.

## Guarantor

Masahide Hiyoshi.

## Declaration of competing interest

None.
